# Industrial‐Scale Seawater Splitting at Engineered Interface of Boron‐Doped Cobalt Sulfide/Metal–Organic Framework Nanosheets Heterostructure

**DOI:** 10.1002/smsc.202500497

**Published:** 2026-03-06

**Authors:** Seyedmahdi Mousavi, Hafiz Adil Qayyum, Muhammad Waqas Khan, Sharafadeen Gbadamasi, Suraj Loomba, Azadeh Nilghaz, Muhammad Haris, Chamali Kaushalya Malaarachchi, Vasundhara Nettem, Anton Tadich, Lars Thomsen, Yongxiang Li, Asif Mahmood, Nasir Mahmood

**Affiliations:** ^1^ School of Engineering RMIT University Melbourne Australia; ^2^ Department of Physics College of General Studies King Fahd University of Petroleum and Minerals Dhahran Saudi Arabia; ^3^ Interdisciplinary Research Center for Hydrogen Technologies and Carbon Management (IRC‐HTCM) King Fahd University of Petroleum and Minerals Dhahran Saudi Arabia; ^4^ School of Science RMIT University Melbourne Australia; ^5^ School of drug delivery, disposition and dynamics Monash University Parkville Australia; ^6^ Australian Synchrotron ANSTO Clayton Australia; ^7^ Centre for Clean Energy Technology School of Mathematical and Physical Sciences Faculty of Science University of Technology Sydney Australia

**Keywords:** ampere‐level stability, electrocatalytic seawater splitting, heterointerface engineering, metal sulfide, metal–organic framework, oxygen evolution reaction

## Abstract

Seawater electrolysis faces several significant obstacles, including low energy efficiency and anode corrosion due to chlorine chemistry, which limit its practical potential. To overcome this, we developed a catalyst composed of boron‐doped CoS_2_ protected by metal–organic framework sheets (MOFs) (B‐CoS_2_/MOF heterostructures). Introducing B atoms into the CoS_2_ layer tunes the surface chemistry to promote adhesion of Ni–MOF. Density functional theory calculations indicate a strong interaction at the heterointerface, with a binding energy of −4.13 eV, where the MOF anchors onto the B‐CoS_2_ surface through a Ni—S bond measuring 2.08 Å, confirming the presence of an ionic bond. This strong heterointerface promotes OH^−^ adsorption while repelling Cl^−^ ions due to the presence of SO_4_^2‐^, effectively mitigating chlorine‐induced degradation. Therefore, the B‐CoS_2_/MOF catalyst achieves an industrial‐scale current density of 1.0 A cm^−2^ at an overpotential of 542 mV in alkaline seawater and operates stably for 600 h, hence suggesting the potential for designing cost‐effective, chlorine‐resistant systems for practical seawater splitting.

## Graphical abstract

Boron‐doped cobalt sulfide protected by the metal‐organic framework sheets (B‐CoS2/MOF heterostructures) enable selective oxygen evolution reaction in alkaline seawater via enhanced interfacial coupling, optimized hydroxide (OH‐) adsorption, and sulfate (SO_4__2_^−^)‐mediated chloride (Cl^−^) repulsion, achieving 1 A cm^−^_2_ at 542 mV with 600 h stability.

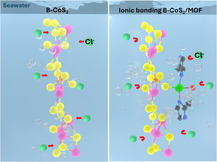

## 1 Introduction

Seawater electrolysis holds great promise as a technology for sustainable and clean hydrogen production, offering a promising alternative to traditional fossil fuels [1, 2]. However, significant challenges must be overcome to make seawater electrolysis viable, such as high energy costs and the need to maintain durability in environments with high chloride ion concentrations [3]. These challenges are especially critical at the anode, where the oxygen evolution reaction (OER) occurs. The chloride ions can also compete with OER at the anode, especially when the overpotential is above 490 mV, producing hypochlorite at pH > 7.5 or chlorine gas at pH < 3 [4, 5, 6].

To achieve enhanced activity and stability under industrial conditions (1 A cm^−2^), the electrocatalysts at the anode should show protection against the Cl^−^ corrosion and prefer the OER.

One common strategy is generating surface‐bound polyanions, such as sulfate (SO_4_^2‐^), which create an electrostatic shield to repel Cl^−^ ions and suppress the competing chlorine evolution reaction (CER) [7]. For instance, a sandwich‐structured NiFe/NiS_*x*_ electrode on nickel foam has been shown to generate SO_4_^2‐^ in situ through oxidation of the underlying NiS_*x*_ layer, effectively inhibiting ClO^−^ formation [8]. Similarly, SO_4_^2‐^ adsorbed on NiFe‐layered double hydroxide surfaces has been reported to enhance corrosion resistance via electrostatic repulsion [8, 9]. However, these surface anions can degrade or leach over long‐term operation, reducing protection. So, embedding such polyanions within robust heterostructures offers a promising way to maintain their presence and function.

The main challenge in developing heterostructures for complex systems, such as seawater, is selecting materials that maintain high activity. Metal–organic frameworks (MOFs), with high surface area, tunable pores, and chemical versatility, are promising for this purpose [10]. However, their low intrinsic OER activity and improper incorporation have limited their use. Recent studies have demonstrated that MOFs, utilized in their original form (without high‐temperature carbonization), can stabilize catalysts and improve efficiency [11, 12, 13]. These studies indicate that forming strong chemical bonds between MOFs and active catalysts is crucial, yet interfacial bonds often corrode under industrial conditions [11, 14]. Therefore, interface engineering is essential to ensure high reaction kinetics, anticorrosion properties, and selective OER in seawater.

In this study, we developed a 2D heterointerface between the sheets of B‐doped CoS_2_ and Ni‐based MOF to address the corrosion and efficiency challenges of seawater electrolysis. Boron doping in CoS_2_ alters its surface chemistry and binding energy (−4.13 eV, calculated by density functional theory (DFT)), creating strong ionic bonds between B‐CoS_2_ and Ni–MOF through Ni–S–Co, with a bond length of 2.08 Å. The B species at the interface favors OH^−^ adsorption, while SO_4_^2‐^ repels Cl^−^ ions, hence making the anode surface selective for OER over CER. This design enables stable (over 600 h) industrial current output (1.0 A cm^−2^) at a very low overpotential of 542 mV. Hence, this work presents a cost‐effective, non‐noble metal‐based catalyst with strong potential for practical seawater electrolysis.

## 2 Results and Discussion

### 2.1 Synthesis and Morphological Analysis

**FIGURE 1:**
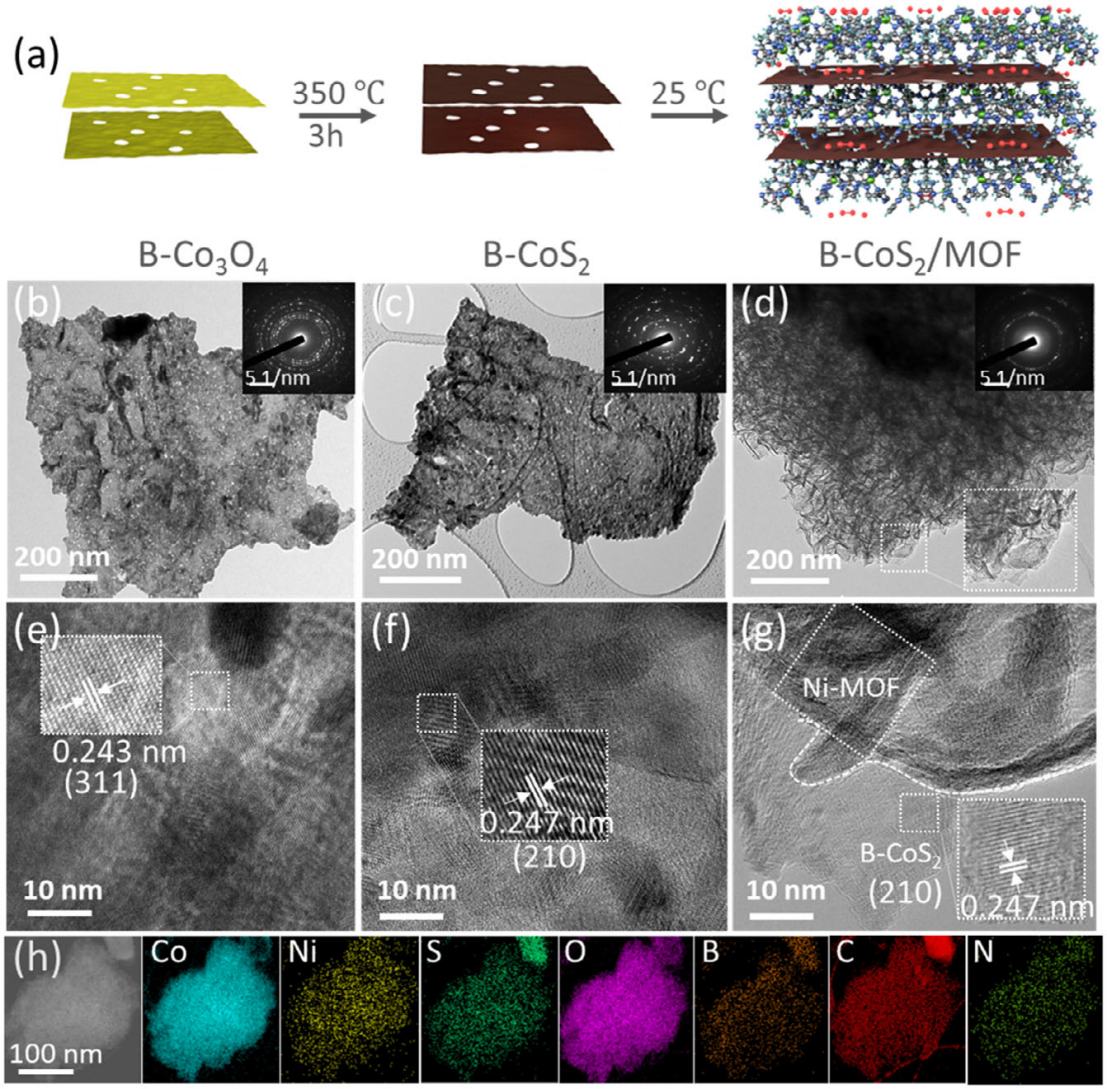
(a) Schematic illustration of B‐CoS_2_/MOF heterostructure. The morphological analysis using TEM (b) B‐Co_3_O_4_, (c) B‐CoS_2_ (d) B‐CoS_2_/MOF (inset: SAED). (e–g) HRTEM images of B‐Co_3_O_4_, B‐CoS_2_, and B‐CoS_2_/MOF. (h) EDS elemental mapping of Co, Ni, S, O, C, N, and B in B‐CoS_2_/MOF. EDS = Energy‐dispersive spectroscopy; HRTEM = high‐resolution transmission electron microscopy; MOF = metal–organic framework; SAED = selected area electron diffraction.

A simple salt‐template method was optimized to grow boron‐doped Co_3_O_4_ nanosheets, followed by thermal annealing. B‐Co_3_O_4_ was then converted to B‐CoS_2_ through the standard sulfurization process. Then, a Ni‐based MOF was grown on B‐CoS_2_ through solid–liquid interfacial chemistry at room temperature, forming a B‐CoS_2_/MOF heterostructure (Figure 1a). The incorporation of B into the CoS_2_ lattice provides assistance for the formation of optimized metal–sulfur bonds at the heterointerface. B doping also introduced the borate species (surface‐associated B–O/BO_*x*_ groups) that preferentially attracted hydroxyl ions at the interface during electrochemical reaction [15]. Transmission electron microscopy (TEM) image of B‐Co_3_O_4_ demonstrated a 2D sheet‐like structure with numerous pore features (Figure 1b), which was preserved at the postsulfuration to obtain B‐CoS_2_ (Figure 1c). It demonstrated the material's morphological stability with porous nanosheet throughout the conversion process. The selected area electron diffraction (SAED) patterns of B‐Co_3_O_4_, B‐CoS_2_, and B‐CoS_2_/MOF (Figure 1b–d, insert) displayed bright rings, revealing polycrystalline diffraction patterns and structures. Additionally, TEM and SEM images of B‐CoS_2_/MOF (Figures 1d & S1) revealed the presence of sheet‐on‐sheet morphology, consistent with the formation of a 2D/2D heterostructure. This architecture is expected to facilitate charge transport and expose more active sites, thereby improving the electrocatalytic performance.

The high‐resolution TEM (HRTEM) image of B‐Co_3_O_4_ (Figure 1e) exhibits a *d*‐spacing of 0.243 nm for the (311) plane, while a *d*‐spacing of 0.247 nm in Figure 1f corresponds to (210) plane of B‐CoS_2_. Similarly, the HRTEM image of B‐CoS_2_/MOF (Figure 1g) shows the presence of a multilayered structure with distinct lattice fringes, having a *d*‐spacing value of 0.247, corresponding to (210) crystallographic plane of B‐CoS_2_. These observations further confirm the successful formation of the 2D/2D B‐CoS_2_/MOF heterostructure [16, 17]. Energy‐dispersive spectroscopy (EDS) mapping reveals the coexistence and uniform distribution of Co, Ni, S, O, C, N, and B elements throughout the B‐CoS_2_/MOF nanosheet (Figures 1h & S2), further confirming the successful synthesis of the B‐CoS_2_/MOF heterostructure.

**FIGURE 2:**
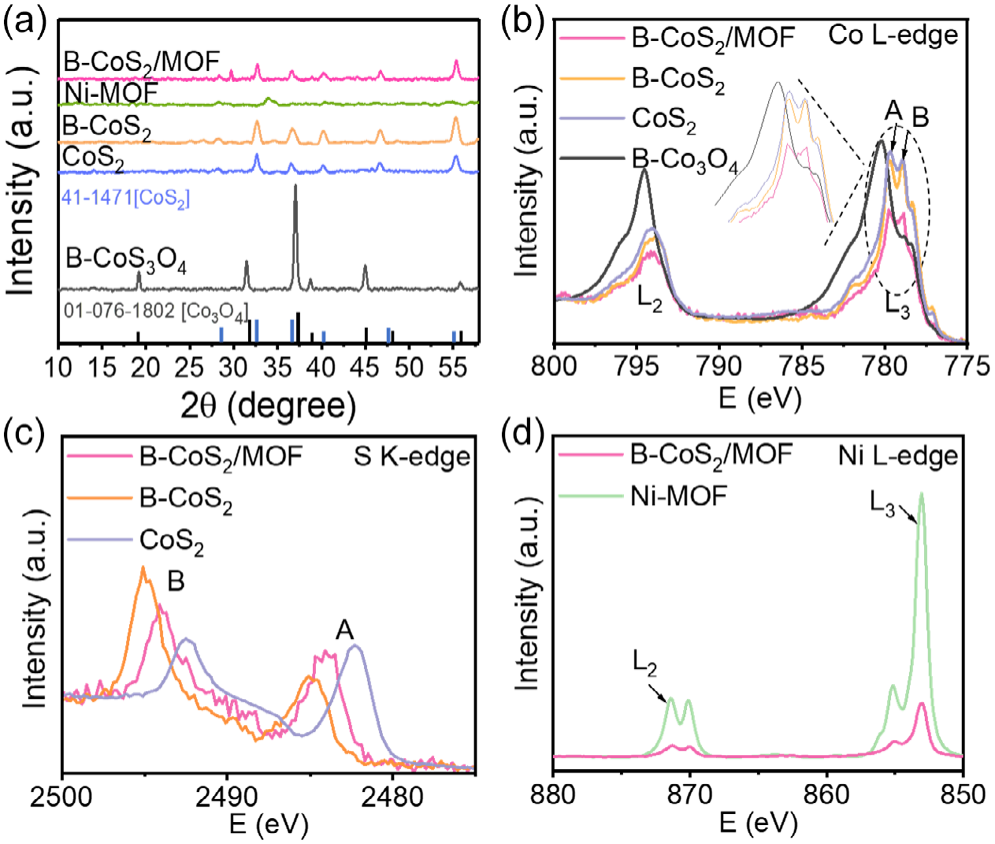
(a) XRD patterns of B‐Co_3_O_4_, CoS_2_, B‐CoS_2_, Ni–MOF, and B‐CoS_2_/MOF. (b) Co L‐edge NEXAFS. (c) S K‐edge NEXAFS, and (d) Ni L‐edge NEXAFS. XRD = X‐ray diffraction.

Figure 2a shows the X‐ray diffraction (XRD) patterns of the synthesized catalysts: B‐Co_3_O_4_, CoS_2_, B‐CoS_2_, Ni–MOF, and B‐CoS_2_/MOF. The distinct diffraction peaks observed across samples confirm their crystalline nature. The peaks for B‐Co_3_O_4_ align well with the standard Co_3_O_4_ reference (PDF 01‐076−1802) and CoS_2_ reference (PDF 14–1471), although slight peak shifts are noted. The shifts are attributed to the incorporation of boron atoms into the B‐Co_3_O_4_ structure. The absence of B‐related peaks implies that B acts as a dopant that modifies the material's electronic structure without affecting the overall bulk crystallinity [18]. The B‐CoS_2_/MOF shows characteristic peaks of both B‐CoS_2_ and the MOF, confirming the existence of both phases. Specifically, the prominent peaks at 2*θ* = 36.9° and 55.6° of B‐CoS_2_ are present in the XRD pattern of B‐CoS_2_/MOF, though with slight shifts due to interfacial electronic interactions and bond formation. Additionally, a peak observed at 2*θ* =17.8° corresponds to the Ni‐based MOF, confirming the presence of the MOF within the heterostructure.

### 2.2 Electronic Structure

The electronic structure and charge states of Co, S, Ni, and O were investigated using synchrotron‐based X‐ray absorption near‐edge spectroscopy (NEXAFS). As shown in Figure 2b, the Co L‐edge spectra are split into the L_3_ and L_2_ edges due to spin–orbit coupling of the 2p core levels [19, 20]. The L_3_ edge exhibits two peaks, at 779.1 and 780.1 eV, indicating consistent cobalt valence states across the different compositions. The peak near 780 eV further splits into two distinct peaks, labeled as “A” and “B,” corresponding to Co^2+^ and Co^3+^ states in CoS_2_, B‐CoS_2_, and B‐CoS_2_/MOF, respectively. These features, including weaker shoulder peaks, exhibit shifts in binding energy, attributed to sulfurization and electrostatic interactions arising from heterostructure formation [21]. In the B‐CoS_2_/MOF heterostructure, the Co valence state is slightly reduced, resulting in a 0.1 eV shift toward lower energy. This shift suggests a lower oxidation state of Co in the Co–S framework, due to charge transfer between Co and Ni atoms mediated by sulfur [22, 23].

The sulfur K‐edge NEXAFS spectra of CoS_2_ (Figure 2c) display two broad absorption features: peak “A” at 2482 eV, associated with the reduction processes and contributions from sulfate (SO_4_^2‐^), and peak “B” at 2492.5 eV, corresponding to disulfide (S_2_^2‐^) species [24]. Upon boron incorporation, both peaks shift to higher energies, indicating structural and electronic modulation, likely due to partial substitution of sulfur atoms by boron. In the B‐CoS_2_/MOF heterostructure, the S_2_^2‐^ peak shifts slightly to a lower energy (2494 eV), showing an approximate 1 eV change compared to B‐CoS_2_. Meanwhile, the sulfate‐related peak at 2484 eV became noticeably broader than in B‐CoS_2_. These spectral variations suggest enhanced electronic interactions and the formation of strong Co–S–Ni linkages, reflecting the altered chemical environment within the heterostructure.

The Ni L‐edge NEXAFS spectrum indicates two main adsorption features (L_3_ and L_2_), each accompanied by subtle shoulder peaks corresponding to Ni^2+^ and Ni^3+^ oxidation states (Figure 2d). In pure Ni–MOF, the Ni^2+^ L_2_ peak appears at 871.4 eV, showing a slight positive shift of 0.1 eV compared to the same peak in the B‐CoS_2_/MOF heterostructure, which appears at 871.3 eV. Additionally, the Ni^3+^ peak at 853 eV shows reduced intensity in the heterostructure, indicating electron transfer from B‐CoS_2_ to Ni–MOF. This electron transfer alters the distribution of Ni oxidation state [17, 25]. The O K‐edge NEXAFS spectra show that oxygen nature changes from metallic bonding to nonmetal interactions such as formation of sulfate and oxygen species in MOF. (Figure S3).

**FIGURE 3:**
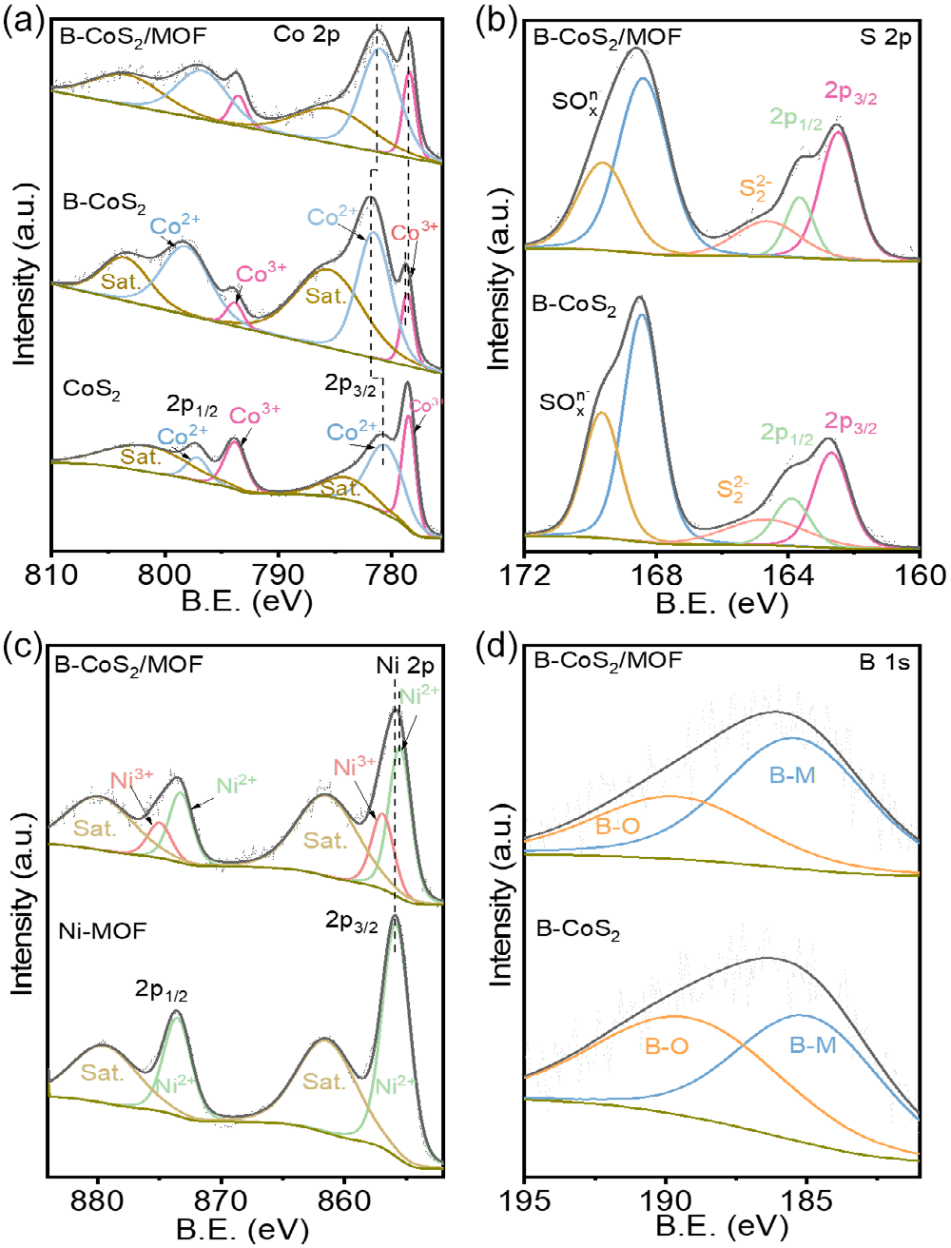
Overall XPS spectrum analysis of B‐CoS_2_ and B‐CoS_2_/MOF. (a) Co 2p, (b) S 2p, (c) Ni 2p, and (d) B 1s core‐level XPS spectra. XPS = X‐ray photoelectron spectroscopy.

The X‐ray photoelectron spectroscopy (XPS) was further used to analyze valence states in CoS_2_, B‐CoS_2_, and B‐CoS_2_/MOF. Figure 3a displays the Co 2p spectra with two distinct Co 2p_3/2_ and Co 2p_1/2_ peaks, indicating the coexistence of Co^2+^ and Co^3+^ in all three samples. The deconvoluted Co 2p spectrum of B‐CoS_2_/MOF shows shifts of −0.5 to −0.2 eV for Co^3+^ and Co^2+^, respectively, compared to B‐CoS_2_. Additionally, an increased intensity of the Co^3+^ signal in B‐CoS_2_/MOF may be attributed to interfacial charge redistribution during the formation of new Co—S—Ni bonds at the heterointerface [26, 27]. The high‐resolution S 2p XPS spectra of B‐CoS_2_ and B‐CoS_2_/MOF (Figure 3b) exhibit peaks at 168.4 and 169.6 eV, attributing to SO_*x*_^n‐^ species formed by surface sulfur oxidation upon air exposure [22]. Peaks at 162.4 and 163.6 eV correspond to the S 2p_3/2_ and S 2p_1/2_ orbitals of S_2_^2‐^ species within the heterostructure, respectively [22]. Additionally, the peak at 164.6 eV suggests the presence of bridging S_2_, indicating that sulfur is involved in the connections between Ni and Co within the heterostructure [28]. The presence of surface sulfate (SO_4_^2‐^) plays a critical role in forming a passivation layer, which protects the surface by repelling chloride anion through electrostatic repulsion, thereby mitigating chloride‐induced corrosion [29, 30].

The Ni 2p spectra of Ni–MOF and B‐CoS_2_/MOF heterostructure reveal two main peaks associated with Ni 2p_1/2_ at 873.5 and 875 eV, and Ni 2p_3/2_ at 855.8 and 856.9 eV (Figure 3c). The Ni^2+^ peaks in B‐CoS_2_/MOF are negatively shifted compared to those in Ni–MOF, implying a higher oxidation state in the heterostructure. The new peaks at 856.9 and 875 eV are attributed to the Ni^3+^ oxidation state, arising from a new ionic interaction formed (Ni–S–Co) at the heterointerface when Ni–MOF is assembled on B‐CoS_2_. The O 1s spectra of the heterostructure were deconvoluted into three peaks at 531.4 and 532.2 eV, corresponding to metal–oxygen (M—O) bonds and M–OH species, and a peak at 532.8 eV indicating adsorbed oxygen in surface hydroxyl groups (H–O) (Figure S4) [31]. Figure 3d shows the high‐resolution B 1s XPS spectrum, featuring two spin–orbit doublets at 188 eV (B—M bonds) and 191.1 eV (B—O bonds) [31].

### 2.3 Theoretical Insights from Density Functional Theory Calculations

**FIGURE 4:**
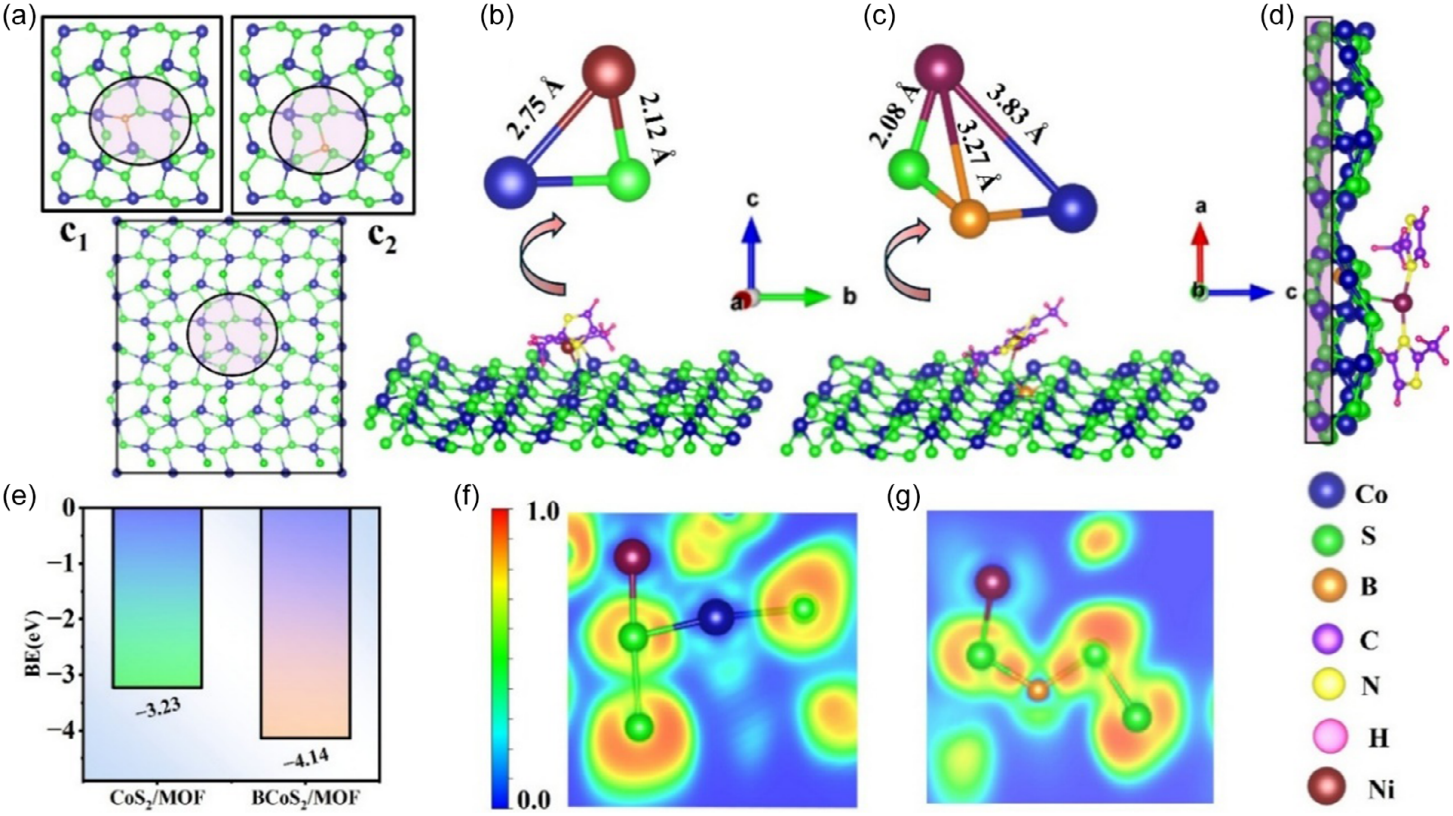
(a) The crystal structure of the CoS_2_ (210) plane along with the substitutional sites c_1_ and c_2_ used for B doping. (b,c) The formation of CoS_2_/MOF and B‐CoS_2_/MOF heterostructures, respectively, along with the distance of Ni–MOF from the nearest atoms of the substrate. (d) Metal sulfide/MOF geometry with the bottom layer in the shaded region was kept fixed in the calculations. (e) The binding energy of the MOF fragment on CoS_2_/MOF and B‐CoS_2_/MOF. (f,g) ELFs of CoS_2_/MOF and B‐CoS_2_/MOF, respectively. ELF = Electron localization function; MOF = metal–organic framework.

The structural and electronic properties of boron‐doped CoS_2_, CoS_2_/MOF, and B‐CoS_2_/MOF were systematically studied using DFT calculations. Regarding B doping in CoS_2_, two possible substitutional sites were considered on the CoS_2_ (210) surface as shown in Figure 4a. In the first configuration (c_1_), an S atom was replaced by a B atom, while in the second configuration (c_2_), a B atom substitutes for a Co atom. The thermodynamic stability of each configuration was assessed by calculating the formation energies using the expression

(1) $$E_{F}=E_{\text{doped}}-E_{\text{pristine}}+{\text{µ}}_{\text{host}}-{\text{µ}}_{B}$$

where $E_{F}$ and $E_{\text{doped}}$ were the total energies of the doped and pristine CoS_2_ surfaces, respectively. ${\text{µ}}_{\text{host}}$ represents chemical potentials of the Co or S atom, while ${\text{µ}}_{B}$ is the chemical potential of boron. Based on the formation energy calculation, the CoS_2_ surface doped with B at the Co site was identified as the most stable configuration, exhibiting a lower formation energy of −0.57 eV, and was used further for the adsorption of the MOF fragment.

Figure 4b and c illustrates the MOF adsorption on CoS_2_ and B‐CoS_2_ surfaces, respectively. For both cases, the MOF was adsorbed at the sulfur site with the Ni—S bond length in B‐CoS_2_/MOF heterostructure being slightly shorter (2.08 Å) compared to that in the CoS_2_/MOF system (2.12 Å). This indicates a stronger interaction between the MOF and the B‐CoS_2_ surface. Further, to quantify the interaction strength, the binding energy ($E_{B}$) was calculated using the following expression

(2) $$E_{B}=E_{\mathrm{Co}S_{2}\left(B-{\mathrm{CoS}}_{2}\right)/\mathrm{MOF}}-E_{\mathrm{Co}S_{2}\left(B-{\mathrm{CoS}}_{2}\right)}-\;E_{\mathrm{MOF}}$$

where $E_{\mathrm{Co}S_{2}(B-{\mathrm{CoS}}_{2})/\mathrm{MOF}}$ is the total energy of the CoS_2_ (B‐CoS_2_)/MOF heterostructure, $E_{\mathrm{Co}S_{2}(B-{\mathrm{CoS}}_{2})}$ is the energy of CoS_2_ (B‐CoS_2_), and $E_{\mathrm{MOF}}$ is the energy of the isolated MOF fragment. Figure 4d shows B‐CoS_2_/MOF geometry, with the bottom layer in the shaded region, which was kept fixed in the calculations. Figure 4e shows the binding energies for the two heterostructures with $E_{B}$. The MOF on B‐CoS_2_ is −4.13 eV, whereas it is −3.23 eV for MOF that binds on the pristine CoS_2_ surface. This result indicates that a stronger interaction exists between the MOF fragment and the boron‐doped CoS_2_ surface as compared to the CoS_2_/MOF heterostructure.

To gain deeper insight into the nature of bonding and charge redistribution at the CoS_2_ (B‐CoS_2_)/MOF interface, the electron localization function (ELF) was analyzed. Figure 4f,g presents the ELF maps for both CoS_2_/MOF and B‐CoS_2_/MOF heterostructures. In both cases, a high degree of electron localization is observed around the sulfur atoms, highlighting their role as an electron‐rich site. This localization becomes even more pronounced upon B incorporation, indicating a redistribution of charge induced by B doping. In contrast, the Ni atom shows relatively delocalized electron density, suggesting that the electrons surrounding Ni are relatively dispersed. This delocalization is further enhanced in the B‐CoS_2_/MOF system, as the electron density around Ni becomes more extended toward the S atom, reflecting relatively stronger electronic interaction between the MOF and the B‐CoS_2_ surface. Further, the low ELF values (<0.5) in the region between Ni and S atoms suggest that the Ni—S bond possesses a predominantly ionic character.

### 2.4 Selective Oxygen Evolution Reaction Electrocatalytic Performance

**FIGURE 5:**
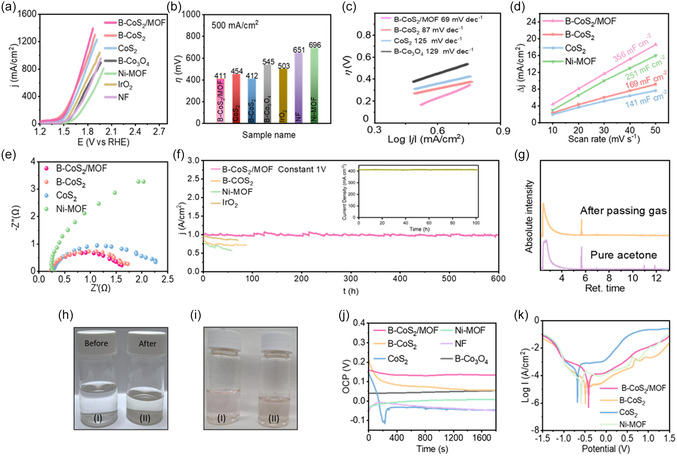
(a) LSV curves of B‐Co_3_O_4_, CoS_2_, B‐CoS_2_, IrO_2_, NF, Ni–MOF, and B‐CoS_2_/MOF for OER in 6 M KOH seawater. (b) The overpotential of different catalysts at 500 mA cm^−2^. (c) Tafel plots obtained from the corresponding polarization curves. (d) ECSA measurements of the B‐CoS_2_, Ni–MOF, and B‐CoS_2_/MOF. (e) EIS spectra of the indicated catalysts. (f) Long‐term stability tests were conducted at a constant current density of 1 A cm^−2^ and a voltage of 1 V in 6 M KOH seawater for various catalysts. The inset shows full‐cell seawater electrolysis stability measured in a zero‐gap electrolzser using B‐CoS_2_/MOF as the anode and commercial Pt/C as the cathode. (g) GC–MS testing of acetone solvent under gas evolved in 2V. (h) The optical image shows an absence of color in the pure acetone both before and after gas was introduced through it for 1 h. (i) DPD test showing that no significant color change in (I) real seawater and (II) electrolyte. (j) OCP curves of Ni–MOF, CoS_2_, B‐CoS_2_, and B‐CoS_2_/MOF in 5 wt% NaCl solution. (k) Potentiodynamic polarization curves of Ni–MOF, CoS_2_, B‐CoS_2_, and B‐CoS_2_/MOF. LSV = Linear sweep voltammetry; OER = oxygen evolution reaction; ECSA = electrochemical surface areas; EIS = electrochemical impedance spectroscopy; GC–MS = gas chromatography–mass spectrometry; OCP = open‐circuit potential.

The OER performance of the B‐CoS_2_/MOF 2D/2D heterostructure and other prepared materials was evaluated in 6 M KOH seawater (Seawater was collected from Altona Beach, Melbourne, Australia) and DI water, and compared with commercial IrO_2_ as a benchmark (Figures 5a & S5). As shown in Figure 5a, linear sweep voltammetry (LSV) curves (without *iR* compensation) indicated that B‐CoS_2_/MOF outperforms other samples (B‐Co_3_O_4_, CoS_2_, B‐CoS_2_, IrO_2_, Ni foam (NF), and Ni–MOF), achieving a current density of 500 mA cm^−2^ at an overpotential of only 411 mV. In contrast, the other catalysts B‐Co_3_O_4_ (545 mV), CoS_2_ (454 mV), B‐CoS_2_ (412 mV), NF (651 mV), Ni–MOF (696 mV), and IrO_2_ (503 mV) require significantly higher overpotentials (Figure 5b). At 542 mV, the B‐CoS_2_/MOF heterostructure achieves a current density of 1 A cm^−2^, significantly outperforming the benchmark IrO_2_, which requires 712 mV to reach the same current density, indicating superior OER performance. Moreover, the B‐CoS_2_/MOF heterostructure exhibited the least Tafel value of 69 mV dec^−1^ (Figures 5c & S6) compared to NF (167 mV dec^−1^), IrO_2_ (159 mV dec^−1^), CoS_2_ (94 mV dec^−1^), B‐Co_3_O_4_ (129 mV dec^−1^), B‐CoS_2_ (87 mV dec^−1^), and Ni–MOF (178 mV dec^−1^), indicating faster OER reaction kinetics [32]. Additionally, the electrochemical double‐layer capacitance (C_dl_) was obtained from the scan‐rate‐dependent cyclic voltammetry (CV) in the non‐Faradaic potential region (Figures 5d & S7) to gain insight into the electrochemical surface areas (ECSA) of the catalysts. As shown in Figure 5d, the B‐CoS_2_/MOF exhibited the highest C_dl_ value of 131.87 mF cm^−2^, correlating to the largest active surface area. Moreover, the electrochemical impedance spectroscopy (EIS) was conducted to further investigate the catalytic kinetics. The Nyquist plots in Figure 5e display that the B‐CoS_2_/MOF has the smallest semicircle diameter among the comparative samples, indicating the highest charge transfer capability and lowest resistance at the electrode/electrolyte interface.

The electrochemical stability of B‐CoS_2_/MOF was evaluated through long‐term stability tests under a constant voltage (1 V vs. Hg/HgO) in alkaline seawater under ambient laboratory conditions (22 ± 3°C). As shown in Figure 5f, the I–t output of the B‐CoS_2_/MOF heterostructure remained stable at 1 A cm^−2^ without noticeable degradation for over 600 h, demonstrating excellent OER durability. Moreover, full‐cell seawater splitting stability was assessed using a zero‐gap electrolyzer in a two‐electrode configuration where, as‐developed B‐CoS_2_/MOF was employed as the anode and commercial Pt/C as the cathode, the system sustained a steady current density of ≈420 mA cm^−2^ for over 100 hr, with no noticeable performance drop as shown in Figure 5f inset. This remarkable stability is mainly attributed to the predominantly ionic connections at the heterointerface, repulsion to the chlorine species and favors to OER. In contrast, a significant current drop was observed after ≈100, 75, and 60 h for B‐CoS_2_, IrO_2_, and Ni–MOF, respectively, with a high degradation rate when tested at a current density of ≈1 A cm^−2^, mainly due to severe Cl^−^‐induced corrosion at the active sites. Table S1 compares the OER performance of B‐CoS_2_/MOF with that of other reported catalysts, highlighting key metrics such as overpotential, Tafel slope, durability, and metal content, thereby providing a clearer context for evaluating the novelty and advantages of this work.

The OER selectivity of B‐CoS_2_/MOF over CER was investigated by passing the gas generated during a continuous electrochemical process at 2 V for 1 h through acetone, left overnight under light. The resulting liquid product was analyzed using gas chromatography–mass spectrometry (GC–MS). Chlorine gas is known to react completely and instantaneously with acetone at room temperature to produce chloroacetone, which develops an amber coloration on exposure to light [33]. The results confirmed no Cl_2_ gas during the process, as no new peak corresponding to chloroacetone was detected (Figure 5g). This is further supported by Figure 5hII, where the solution remains colorless as pure acetone even after light exposure, implying the absence of chloroacetone. These findings indicate that the heterointerface selectivity favors OER while effectively inhibiting the CER. The catalyst's selectivity for OER was further confirmed by calculating the Faradaic efficiency of B‐CoS_2_/MOF. At a constant current density of 1.9 A cm^−2^ and an applied potential of 2 V, the generated O_2_ reached a Faradaic efficiency of 91%, signifying that electron transfer was mainly driven by the OER (SI note 1). The N, N‐diethyl‐p‐phenylenediamine (DPD) reagent was also used to detect hypochlorite (ClO^−^) generation during seawater splitting. For each test, 2 µL of DPD solution was added to 20 mL of real seawater and alkaline seawater, with the pH adjusted to 7.0. In alkaline seawater, the reagent developed a light pink color, indicating the presence of hypochlorite. However, a much lighter color was observed in the testing electrolyte Figure 5iII, indicating negligible ClO^−^ formation after testing at a cell voltage of 1.72 V. To probe the fate of SO_4_^2‐^ ion during seawater electrolysis, spectrophotometric analysis was performed on the spent electrolyte after 1 hr of continuous operation at 2.0 V versus RHE, with filtered seawater used as a control. The measured SO_4_^2‐^ ion concentrations for B‐CoS_2_/MOF, CoS_2_, and filtered seawater were 8, 8, and 7 ppm, respectively. The negligible difference relative to the control indicates the absence of detectable SO_4_^2‐^ leaching during operation, supporting the retention of SO_4_^2‐^ ion at the electrode–electrolyte interface. This behavior is consistent with effective suppression of chloride access to active sites, thereby mitigating competing CER.

### 2.5 Anticorrosion Performance and Selectivity Mechanism

To investigate the corrosion resistance of the catalysts in seawater electrolysis, their corrosion performance was assessed using open‐circuit potential (OCP), which reflects the corrosion behavior (Figure 5j). The higher value and stable curve of the B‐CoS_2_/MOF indicate that the ionically bonded heterostructure quickly forms an anticorrosive protective layer on the surface to avoid corrosion. Figure 5k displays the polarization curves of various catalysts after the 30 min stabilization period. According to Table S2, the B‐CoS_2_/MOF electrode showed a less negative corrosion potential (*E*_corr_) of −0.41 V as compared to B‐CoS_2_ (−0.48 V), CoS_2_ (−0.67 V), and Ni–MOF (−0.58 V). It indicates that B‐CoS_2_/MOF is more stable and less prone to corrosion as compared to other catalysts. The corrosion rate of B‐CoS_2_/MOF (0.848 cm/year) is also lower compared to the other samples.

**FIGURE 6:**
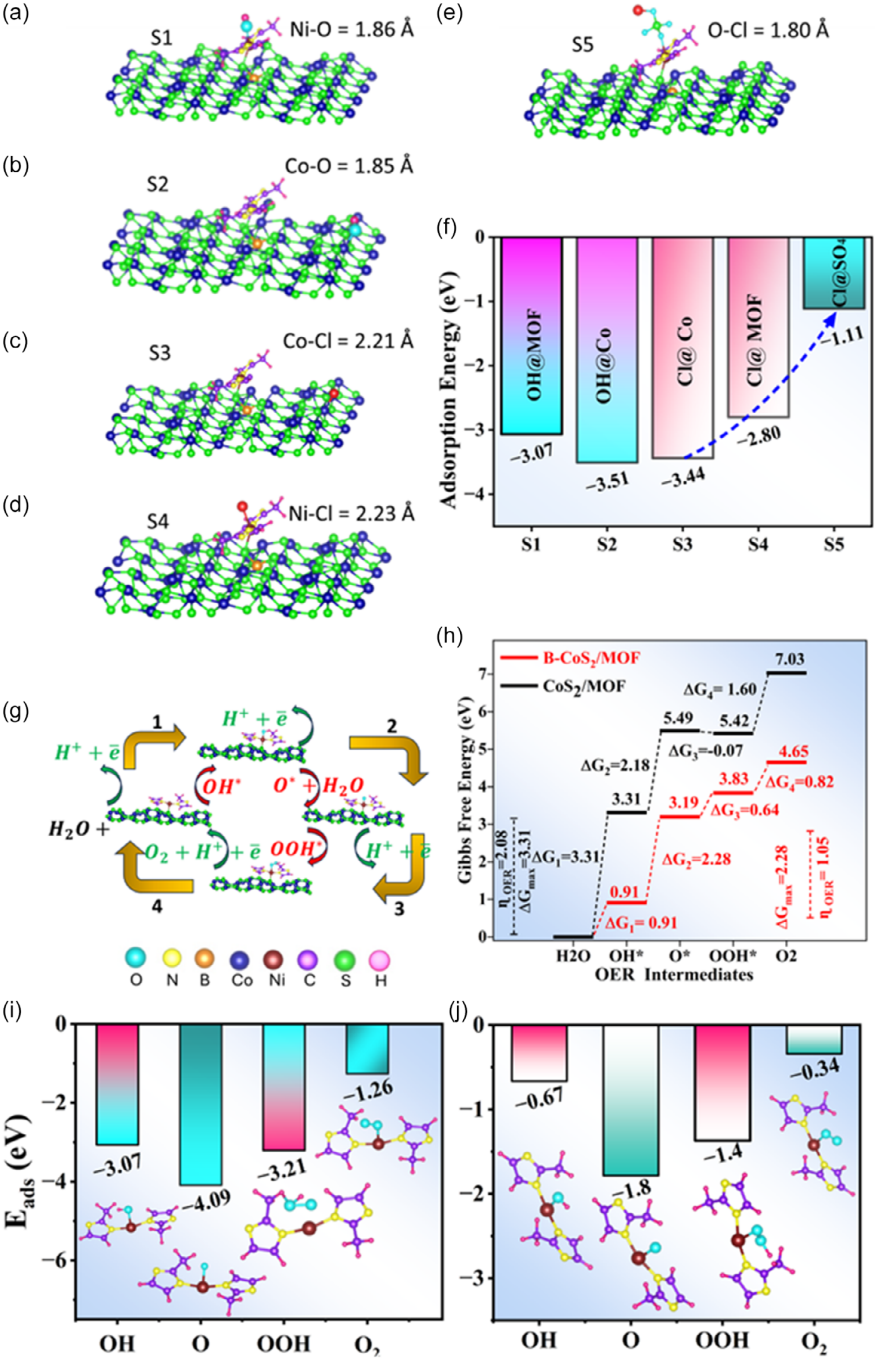
Adsorption feature of OH over the surface of MOF (a) and (b) bare B‐CoS_2_. (c,d) Cl adsorption at the bare B‐CoS_2_ site and the MOF site, respectively. (e) Cl adsorption on SO_4_ passivated B‐FeS_2_/MOF structure. (f) The adsorption energies of OH and Cl ions at different sites of the catalyst. (g) Schematic representation of OER pathways. (h) Gibbs free energy diagram for OER using CoS_2_/MOF and B‐CoS_2_/MOF catalysts. (i,j) The adsorption energies of the intermediates (along with their adsorption sites at the MOF) involved in OER using B‐CoS_2_/MOF and CoS_2_ /MOF catalysts, respectively. The oxygen and chlorine atoms are shown in cyan and red colors, respectively. MOF = Metal–organic framework; OER = oxygen evolution reaction.

Moreover, B‐CoS_2_/MOF's preference for the OER over the CER is due to the rapid formation of a protective passivation layer on its surface. This layer helps to repel chloride ions, driven by electrostatic repulsion from negatively charged polyanions. To evaluate the catalyst selectivity under seawater electrolysis conditions, the competitive adsorption behavior of hydroxyl (OH*) and chloride (Cl*) species on the B‐CoS_2_/MOF surface was systematically investigated, as shown in Figure 6a–f. For each species, two primary adsorption sites were considered. One is at the MOF site (S1 and S4, respectively, associated with OH^*^ and Cl^*^ adsorption at the MOF site), while the second one is at the bare CoS_2_ site (S2 and S3 associated with OH* and Cl* adsorption at the bare CoS_2_). For OH*, the B‐CoS_2_/MOF exhibits significantly stronger adsorption at both S1 and S2 sites. For example, when OH* is adsorbed at the Ni site of the MOF, the adsorption energy is calculated to be −3.07 eV, accompanied by the formation of a Ni—O bond with a bond length of 1.86 Å. Similarly, when OH* is adsorbed at the bare CoS_2_ site, an even stronger adsorption energy of −3.51 eV is observed, with the resulting Co—O bond length of 1.85 Å. The high adsorption energy values indicate that the MOF and the bare B‐CoS_2_ surface are both catalytically active toward OER. However, the existence of MOF shields the B‐CoS_2_ surface from the possible chlorine attack, which ultimately prevents corresponding catalyst degradation. This is evident by the fact that the adsorption energy of Cl* adsorbed at the Co site of B‐CoS_2_/MOF surface is −3.44 eV with a Co—Cl bond length of 2.21 Å. In contrast, the adsorption of Cl* becomes less favorable at the Ni position of MOF (Ni—Cl bond length of 2.23 Å) with adsorption energy dropping to −2.80 eV.

Additionally, the presence of the SO_4_^2‐^ layer further suppresses Cl* anchoring on the catalyst surface as the adsorption energy is further decreased to −1.11 eV, as shown in Figure 6f, when Cl* was adsorbed at the B‐CoS_2_/MOF catalyst passivated with SO_4_^2‐^ layer. These findings confirm that B‐CoS_2_/MOF, along with SO_4_^2‐^ passivation, not only promotes efficient OER activity by preferentially adsorbing OH* intermediates but also exhibits robust resistance to chlorine‐induced corrosion and side reactions, making it a promising candidate for durable and selective seawater electrolysis.

### 2.6 Reaction Pathways of Oxygen Evolution Reaction

The OER activity of CoS_2_/MOF and B‐CoS_2_/MOF heterostructures was investigated using DFT calculations. Figure 6g shows the illustration of the OER mechanism, which proceeds via the following four elementary steps

(3) $$H_{2}O+\text{*}\;\to \;H^{+}+\overset{―}{e}+{\mathrm{OH}}^{*}$$

(4) $${\mathrm{OH}}^{*}\;\to \;O^{*}+H^{+}+\overset{―}{e}$$

(5) $$H_{2}O+O^{*}\;\to \;{\mathrm{OOH}}^{*}+H^{+}+\overset{―}{e}$$

(6) $${\mathrm{OOH}}^{*}\;\to \;O_{2}+\text{*}+\;H^{+}+\overset{―}{e}$$

where ∗ denotes the active site either on the CoS_2_/MOF or on B‐CoS_2_/MOF catalyst surface, and ${\mathrm{OH}}^{*},\;O^{*},$ and ${\mathrm{OOH}}^{*}$were the OER intermediates. The corresponding Gibbs free energy change for each reaction step (under standard conditions) was calculated using the expression.

(7) $$\text{Δ}G=G_{M}+E_{\mathrm{ZPE}}-\text{T}\text{Δ}\text{S}\;$$

where $G_{M}$ was the reaction energy calculated using the expression described in reference [8], $E_{\mathrm{ZPE}}$ was the zero‐point energy, and ΔS was the entropy contribution obtained at a temperature of 298.15 K from nonimaginary frequency calculation of the intermediates.

Figure 6h shows the Gibbs free energy diagram for the OER on CoS_2_/MOF and B‐CoS_2_/MOF catalysts. Here, the rate‐determining step was the one that involves the highest Gibbs free energy between the two consecutive steps ($\text{Δ}G_{\max\;}=\max[\text{Δ}G_{1},\text{Δ}G_{2},\text{Δ}G_{3},\text{Δ}G_{4}]$). Moreover, the overpotential required to drive OER beyond the thermodynamic threshold of 1.23 V was calculated using the expression ($\eta_{\mathrm{OER}}=\frac{\text{Δ}G_{\max}}{e}\;-1.23$). For the pristine CoS_2_/MOF system, the first step, involving the formation of hydroxyl intermediate from H_2_O, constitutes the rate‐determining step exhibiting a substantial energy barrier (Δ*G*_1_ = Δ*G*_max_) of 3.31 eV with an overpotential value (${\text{η}}_{\mathrm{OER}}$) of 2.08 V. Contrarily, the B‐CoS_2_/MOF catalyst shows a dramatically reduced energy barrier for the same step with Δ*G*_1_ = 0.91 eV. Moreover, the rate determining step for the B‐CoS_2_/MOF catalyst has been shifted to the 2^nd^ step, involving OH* → O^*^ transition having a maximum Gibbs free energy change value of 2.28 eV, with significantly lowered overpotential to 1.05 V as compared to that of CoS_2_/MOF.

In addition, to elucidate the stability of the reaction intermediates on the surface of the catalysts during the OER, the adsorption energy of OH*, O^*^, OOH*, and O_2_ was calculated as shown in Figure 6i,j. For the B‐CoS_2_/MOF, the OER intermediates exhibited strong adsorption on the surface of the catalyst with adsorption energies significantly more negative across all intermediates (−3.07 eV for OH*, −4.09 eV for O^*^, −3.21 eV for OOH*, and −1.26 eV for O_2_). In contrast, the undoped CoS_2_/MOF exhibits comparatively weaker adsorption ( −0.67 eV for OH*, −1.80 eV for O^*^, −1.40 eV for OOH*, and −0.34 eV for O_2_), suggesting comparatively less favorable adsorption. Hence, B‐CoS_2_/MOF is likely the better OER catalyst because it provides much stronger stabilization of the key OER intermediates.

### 2.7 Ex Situ Analysis of Heterostructure

**FIGURE 7:**
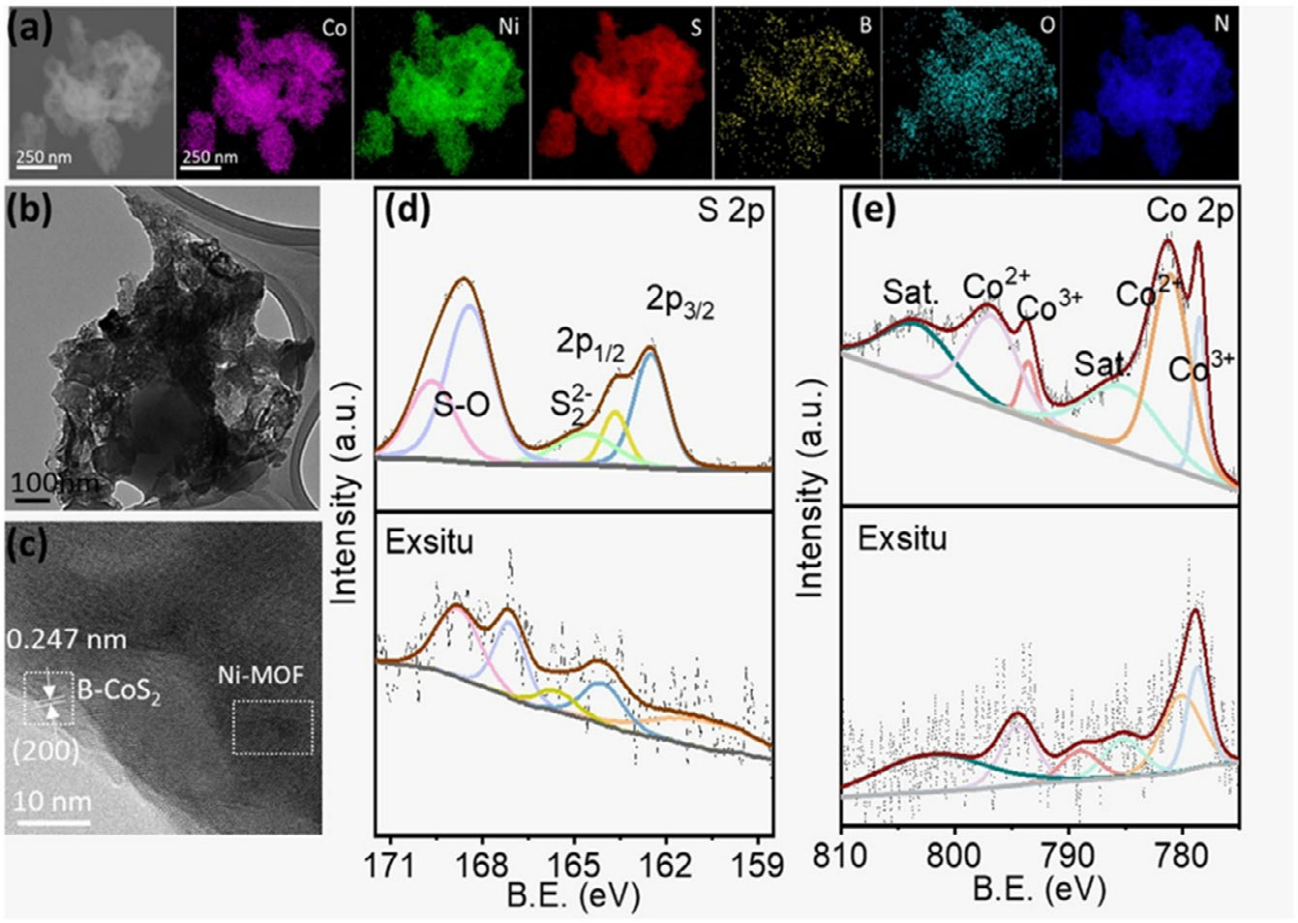
(a) EDS mapping of B‐CoS_2_/MOF after stability testing. (b) TEM image of B‐CoS_2_/MOF. (c) HRTEM of heterostructure. (d,e) XPS deconvoluted ex situ of S and Co. EDS = Energy‐dispersive spectroscopy; TEM = transmission electron microscopy; HRTEM = high‐resolution transmission electron microscopy; XPS = X‐ray photoelectron spectroscopy.

Postelectrochemical (ex situ) testing was conducted to investigate the effects of electrocatalytic testing on the morphology and structure of the strongly bonded B‐CoS_2_/MOF heterostructure. The ex situ EDS elemental mapping of B‐CoS_2_/MOF (Figure 7a) demonstrates that Co, Ni, S, B, O, and N are consistently distributed throughout the heterostructure after electrochemical testing, with no foreign contaminants present. The TEM image (Figure 7b) and SEM image (Figure S8) show that the overall morphology of the heterostructure was retained after electrochemical testing, while HRTEM (Figure 7c) further supports this, revealing a lattice spacing of 0.247 nm, corresponding to the (200) plane of CoS_2_ (JCPDS No. 41–1471). Furthermore, the deconvoluted XPS spectra of S 2p (Figure 7d) and Co 2p (Figure 7e) confirm the chemical stability of the catalyst. Notably, the Co^2+^ peak in the Co 2p_3/2_ region exhibits a slight positive shift to 781 eV, suggesting changes in the oxidation state resulting from electrochemical activation. The Raman spectra (Figure S9) show that the characteristic sulfur‐associated vibrational features of the catalyst are well preserved after testing. The peak around 380 cm^−1^ is typically associated with the vibrational modes of Co—S bonds. These ex situ results and theoretical studies confirm the structural robustness and chemical integrity of the B‐CoS_2_/MOF heterostructure as a stable and effective electrocatalyst for industrial‐scale anodic reactions.

## 3 Conclusion

To sum up, we developed an efficient strategy to engineer the interface of B‐CoS_2_/MOF heterostructure through introducing B and SO_4_^2‐^ species to strengthen the heterinterface for selective anodic reaction in the seawater. B tunes the surface binding energy to −4.13 eV, where the Ni–MOF anchors onto the B‐CoS_2_ surface through a Ni—S bond measuring 2.08 Å . Further, B species play a critical role in hydrolyzing the interface through B‐OH and assist SO_4_^2‐^ species to repel chloride ions for high selectivity. It resulted in exceptional OER activity where B‐CoS_2_/MOF required very low overpotentials of 542 mV to deliver the current densities of 1 A cm^−2^. The formation of strong interfacial Co—S—Ni bonds not only enriched the density of active sites but also suppressed cobalt leaching and enhanced selectivity toward OER over the competing CER. This interfacial network maintained high conductivity and contributed to exceptional operational stability, with the B‐CoS_2_/MOF remaining active for over 600 h in seawater at a current density of more than 1 A cm^−2^. DFT confirmed the catalyst's intrinsic selectivity and corrosion resistance by showing strong OH adsorption and suppressed Cl binding, particularly with SO_4_^2‐^ passivation. This work offers a durable and scalable anodic platform for practical seawater electrolysis. Future studies will extend this work to flow‐cell testing by integrating with newly developed or commercially available cathode catalysts.

## 4 Experimental Section

### 4.1 Synthesis of B‐CoS_2_

The precursor solution was prepared by dissolving 300 mg of Co(NO_3_)_2_·6H_2_O, 20 mg of boric acid, and 10 mL of ethanol, followed by stirring with a magnetic stirrer for 20 min. To synthesize cobalt oxyhydroxide, the precursor solution was added to 31 g of NaCl in a 300 mL glass beaker, serving as a salt‐based template. The mixture was then heated at 80°C for 30 min during the mixing step to ensure homogeneous mixing. Subsequently, the precursor@template system was incubated for 2 days to allow the growth process to complete.

The resulting precursor@template system was annealed to form metal oxides, which created nanosheets on the template. The precursor@template was placed in a quartz crucible and heated in a tube furnace under a nitrogen (N_2_) atmosphere at 350°C for 3 h, with a heating rate of 2°C/min. Upon completion, the metal oxides were washed three times with deionized water to remove the NaCl templates, followed by filtration. The final product was collected and redispersed in absolute ethanol. The same annealing procedure was applied to the cobalt oxyhydroxide materials in the presence of sulfur to synthesize B‐CoS_2_.

### 4.2 Synthesis of Metal–Organic Framework and Heterostructure

B‐CoS_2_/MOF was synthesized using an easy approach. Initially, 100.5 mg of Ni(NO_3_)_3_·6H_2_O was dissolved in 22 mL of deionized water. In a separate step, a solution of the 2‐methylimidazole linker was prepared using 22 mL of deionized water. Subsequently, 15 mg of B‐CoS_2_ was dispersed in a mixture of 29.3 mL of methanol and 14.7 mL of deionized water (in a 2:1 ratio) in a beaker, followed by sonication for 1 h. After sonication, the two precursor solutions were added to the B‐CoS_2_ dispersion and allowed to react for 2 h at room temperature.

Note: The details of chemicals and characterization methods are provided in the supplementary information document.

## Supporting Information

Additional supporting information can be found online in the Supporting Information section. **Supporting Figure S1.** SEM image of the B‐CoS_2_/MOF heterostructure. The circled region highlights a sheet‐on‐sheet morphology. **Supporting** **Figure S2.** EDS spectrum of B‐CoS_2_/MOF heterostructure. **Supporting** **Figure S3.** O K‐edge NEXAFS of B‐Co_3_O_4_, CoS_2_, B‐CoS_2_, Ni–MOF, and B‐CoS_2_/MOF heterostructure. **Supporting** **Figure S4.** XPS spectra of O 1s, and N 1s of B‐CoS_2_, B‐CoS_2_/MOF heterostructure. **Supporting** **Figure S5.** LSV curves of various catalysts in 6 M KOH DI water. **Supporting** **Figure S6.** Tafel plots obtained from the corresponding polarization curves. **Supporting** **Figure S7.** CV curves of Ni–MOF, B‐CoS_2_, B‐CoS_2_/MOF heterostructure. **Supporting** **Figure S8.** SEM image of the B‐CoS_2_/MOF heterostructure after high‐current‐density electrochemical testing. **Supporting** **Figure S9.** Raman Spectra before and after electrochemical testing. **Supporting** **Table S1.** Comparison of the OER performance of B‐CoS_2_/MOF with other reported catalysts. **Supporting** **Table S2.** Corrosion testing of different electrodes in 5% NaCl DI water electrolyte.

## Author Contributions

**Seyedmahdi Mousavi**: writing‐original draft, conceptualization, methodology, formal analysis, and data curation. **Hafiz Adil Qayyum**: writing‐review and editing, software, methodology, investigation, formal analysis. **Muhammad Waqas Khan**: writing‐review and editing, supervision, conceptualization, methodology, formal analysis, and data curation. **Sharafadeen Gbadamasi, Suraj Loomba, Azadeh Nilghaz**, **Muhammad Haris, Chamali Kaushalya Malaarachchi, Vasundhara Nettem, Anton Tadich and Lars Thomsen**: formal analysis and data curation. **Yongxiang Li and Asif Mahmood**: writing‐review and editing. **Nasir Mahmood**: writing‐review and editing, supervision, conceptualization, investigation, validation, and project administration.

## Conflicts of Interest

The authors declare no conflicts of interest.

## Supporting information

Supplementary Material

## Data Availability

The data that support the findings of this study are available from the corresponding author upon reasonable request.
