# Supplementary material for: Industrial‐Scale Seawater Splitting at Engineered Interface of Boron‐Doped Cobalt Sulfide/Metal–Organic Framework Nanosheets Heterostructure
Source: Small Sci. 2026 Mar 6;6(3):e202500497. doi: 10.1002/smsc.202500497 (PMC12970157; doi:10.1002/smsc.202500497)
Supplement: Supplementary file 1 — Supplementary Material [file SMSC-6-e202500497-s001.pdf]

Industrial seawater splitting at engineered interface of boron-doped cobalt sulfide/MOF nanosheets heterostructure

Seyedmahdi Mousavi[1], Hafiz Adil Qayyum[2,3], Muhammad Waqas Khan[1,4*], Sharafadeen Gbadamasi[4], Suraj Loomba[4], Azadeh Nilghaz[5], Muhammad Haris[1], Chamali Kaushalya Malaarachchi[4], Vasundhara Nettem[4], Anton Tadic[6], Lars Thomsen[6], Yongxiang Li[1], Asif Mahmood[7] and Nasir Mahmood[1,4*]

1) School of Engineering, RMIT University, Melbourne, VIC 3000, Australia

2) Department of Physics, College of General Studies, King Fahd University of Petroleum and Minerals, Dhahran 31261, Saudi Arabia.

3) Interdisciplinary Research Center for Hydrogen Technologies and Carbon Management (IRC-HTCM), King Fahd University of Petroleum and Minerals, Dhahran 31261, Saudi Arabia.

4) School of Science RMIT University Melbourne, VIC 3000, Australia

5) School of drug delivery, disposition, and dynamics, Monash University, Parkville, VIC 3052, Australia

6) Australian Synchrotron ANSTO, 800 Blackburn Road, Clayton, VIC 3168, Australia

7) Centre for Clean Energy Technology, School of Mathematical and Physical Sciences, Faculty of Science, University of Technology Sydney, NSW 2007, Australia

E-mail:nasir.mahmood@rmit.edu.au; muhammad.waqas.khan@rmit.edu.au

Experimental Methods

Chemicals: Cobalt nitrate nonahydrate, $Co(NO_3)_3.6H_2O$ (Chem-Supply), Nickle nitrate nonahydrate, $Ni(NO_3)_3.6H_2O$ (Chem-Supply), Sodium chloride, 2 methylimidazole (99%, Sigma-Aldrich), NaCl (≥99%, Sigma-Aldrich), Sulfur (99%, Sigma-Aldrich), Boric acid (99%, Sigma-Aldrich), Polytetrafluoroethylene, PTFE (60wt%, Sigma-Aldrich), Potassium hydroxide (KOH), Carbon black, Methanol, Ethanol absolute, and Nickel foam. All chemicals were used as received without any further purification.

Material Characterization: The morphologies of the B-CoS_2/MOF samples were analyzed using transmission electron microscopy (TEM) and high-resolution transmission electron microscopy (HR-TEM) with a JEOL-2100F TEM operating at 200 kV. TEM samples were prepared by drying a droplet of carbon suspension on a copper grid. X-ray photoelectron spectroscopy (XPS) was performed on a Thermo Fisher X-ray photoelectron spectrometer equipped with aluminum radiation as the probe, under a chamber pressure of 5 x 10^{-9} torr. The source power was set to 72 W, utilizing pass energies of 200 eV for survey scans and 50 eV for high-resolution scans, with an analysis spot size of 400 µm in diameter. X-ray diffraction (XRD) analysis was conducted using a Bruker AXS D4 Endeavor. All electrochemical measurements were carried out using a CHI 710D electrochemical workstation. Near-Edge X-ray Absorption Fine Structure (NEXAFS) data were collected at the High Throughput NEXAFS endstation at the Soft X-ray beamline of the Australian Synchrotron, utilizing Partial Electron Yield (PEY) mode with a retarding grid-based channeltron detector, calibrated to an appropriate bias for each element. Gas chromatography was performed using a Varian Gas Chromatograph coupled with a single quadrupole mass spectrometer (GC-MS).

Preparation of Working Electrode: Electrochemical analysis of the samples was carried out in a 6.0 M KOH seawater electrolyte under standard ambient temperature conditions using a CHI 760 D electrochemical workstation (CH Instruments). A saturated Hg/HgCl electrode served as the reference, while a graphite rod was employed as the counter electrode. The working electrode was fabricated by drop-casting the catalyst material onto nickel foam, which had been pre-cleaned with 1 M dilute hydrochloric acid followed by deionized water rinsing. For comparison, polarization curves were also recorded on bare nickel foam without applying iR compensation.

To prepare the catalyst ink, 20 mg of carbon black was dispersed in 20 mL of a 4:1 isopropyl alcohol (IPA) to deionized water mixture and sonicated for 1 hour to achieve uniform dispersion. Then, 5 mg of the B-CoS_2/MOF catalyst was added to 1 mL of this carbon slurry

and subjected to an additional 20 minutes of sonication. Following this, 8 µL of polytetrafluoroethylene (PTFE) binder was introduced, and the mixture was further sonicated for 20 to 30 minutes to ensure homogeneity. Finally, 200 µL of the prepared ink was drop-cast onto a 0.25 cm^2 area of nickel foam and dried in an oven at 50°C.

Electrochemical measurements: Electrochemical assessments were conducted in 6 M KOH seawater under ambient temperature and pressure conditions using a CHI 760 D electrochemical workstation. A saturated mercury-mercury oxide (Hg/HgO) electrode was used as the reference electrode, while a graphite rod functioned as the counter electrode. The working electrode consisted of nickel foam, which was thoroughly cleaned with diluted hydrochloric acid and deionized water prior to use. Polarization curves were recorded at a scan rate of 5 mV/s without iR compensation.

To prepare the catalyst ink, the powdered sample was mixed with a carbon black solution in a 4:1 ratio. Subsequently, a PTFE solution (60% in water, Sigma) was added, and the mixture was sonicated to form a homogeneous ink. The carbon black solution was prepared by dispersing 20 mg of carbon in 20 mL of a 4:1 isopropyl alcohol–water mixture, followed by 1 hour of sonication. Finally, 200 µL of the prepared ink was drop-cast onto a 0.25 cm^2 area of the nickel foam electrode.

To assess the electrochemical double-layer capacitance (C_{dl}), cyclic voltammetry (CV) measurements were conducted across various scan rates (10, 20, 30, 40, and 50 mV s^{-1}). Electrochemical impedance spectroscopy (EIS) was performed over a frequency range of 1 to 10,000 Hz using an AC voltage with an amplitude of 5 mV. Stability tests were executed at a fixed potential to evaluate performance over time. Full-cell seawater splitting stability was assessed at a cell voltage of 1.23 V using a zero-gap electrolyser in a two-electrode configuration. The configuration is 5 x 5, and the active area is 25 cm^2. AutoLab equipped with 10 A booster was used for the full-cell seawater splitting.

Computational details: Theoretical calculations, within the framework of density functional theory (DFT), were performed using the Vienna Ab-initio Simulation Package (VASP) [1–3]. The generalized gradient approximation (GGA) with the Perdew-Burke-Ernzerhof (PBE) functional scheme was used to treat the exchange-correlation interactions [4]. A Hubbard U correction with U values of 3.0 eV and 2.5 eV was employed to describe the strongly correlated d orbitals of Co and Ni atoms respectively [5]. Moreover, Grimme’s DFT-D3 dispersion correction method was incorporated to describe the long-range Van der Waals (vdW)

interactions [6]. A plane-wave energy cutoff with the value of 550 eV was used in all calculations along with a gamma-centered 3×3×1 k-point mesh for both structural and the subsequent self-consistent field (SCF) calculations. Geometry optimizations were carried out until the Hellman−Feynman force on each relaxed atom was less than -1 x 10^{-3} eV/Å, and the total energy of the system was converged to 1 x 10^{-5} eV.

The heterostructure models were constructed by adsorbing a unit of Ni based ZIF-67 metal organic framework (MOF) onto the pristine and boron-doped CoS_2 substrates. For this purpose, a (210) plane associated with CoS_2, as predicted in the XRD analysis, was cleaved and further extended up to [4 × 2] supercell to accommodate the size of the MOF fragment. The MOF fragment was initially placed 3 Å above the surface. During relaxation, the bottom atomic layers of the slab were kept fixed. The spurious interactions between periodic images were eliminated by introducing a vacuum of 20 Å in the out-of-plane (z) direction. Post-processing and analysis of the VASP output files were conducted using the VASPKIT code [7].

Results

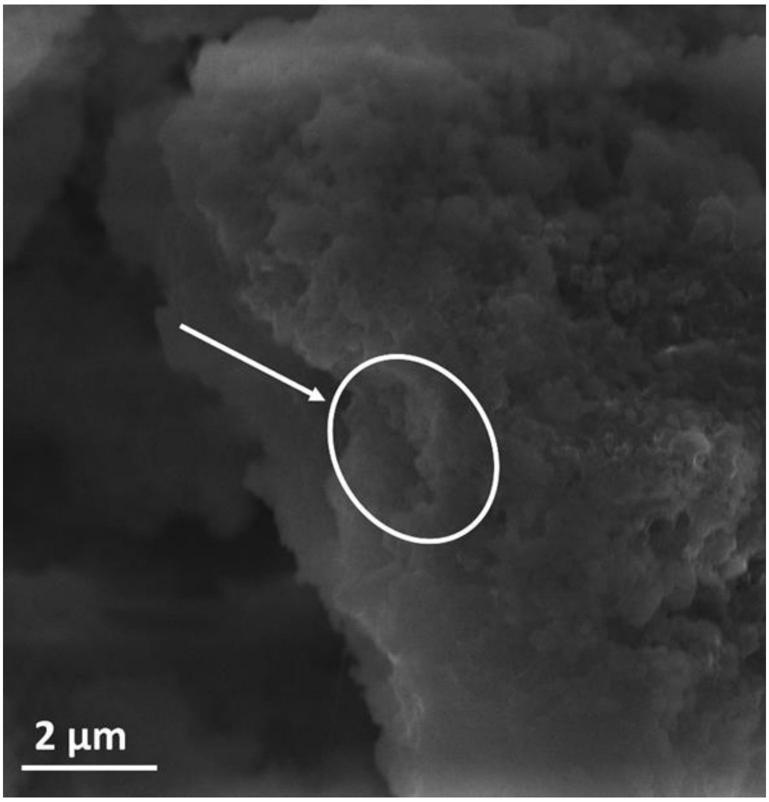

Figure S1. SEM image of the B-CoS_2/MOF heterostructure. The circled region highlights a sheet-on-sheet morphology.

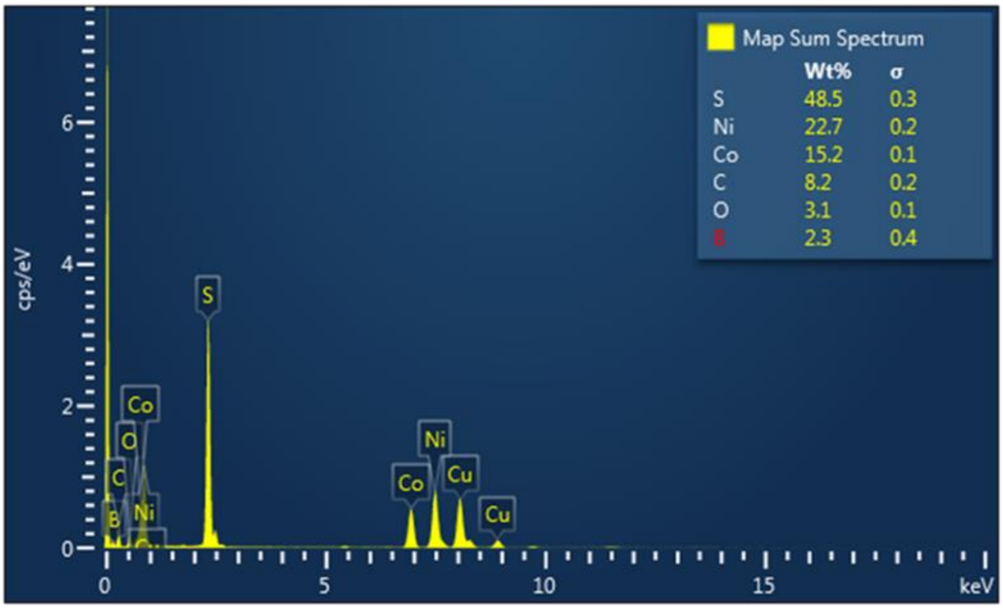

Figure S2. EDS spectrum of B-CoS_2/MOF heterostructure.

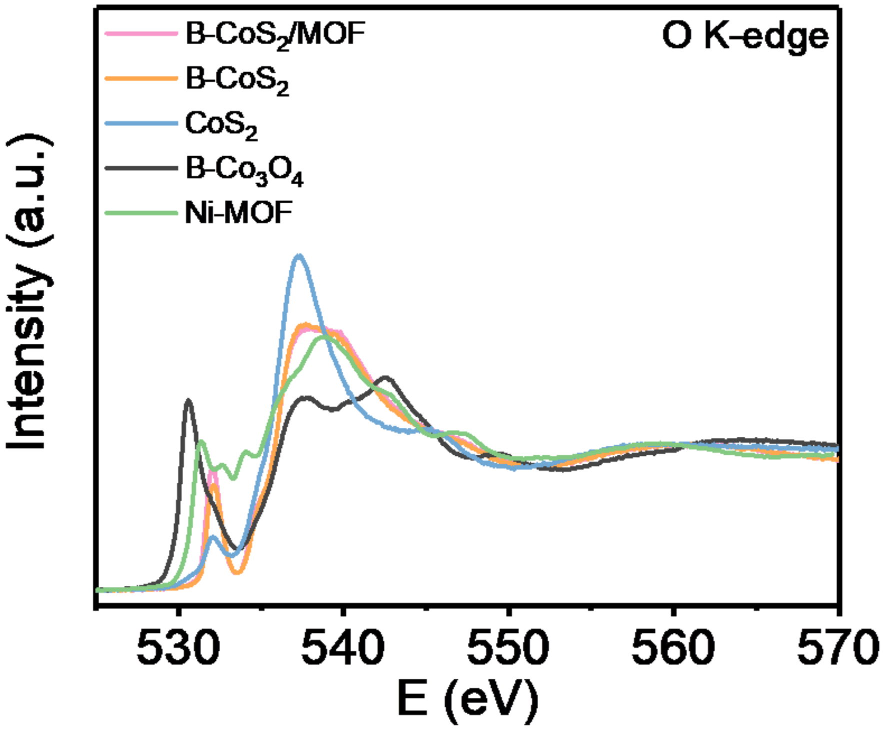

Figure S3. O K-edge NEXAFS of B-Co_3O_4, CoS_2, B-CoS_2, Ni-MOF and B-CoS_2/MOF heterostructure.

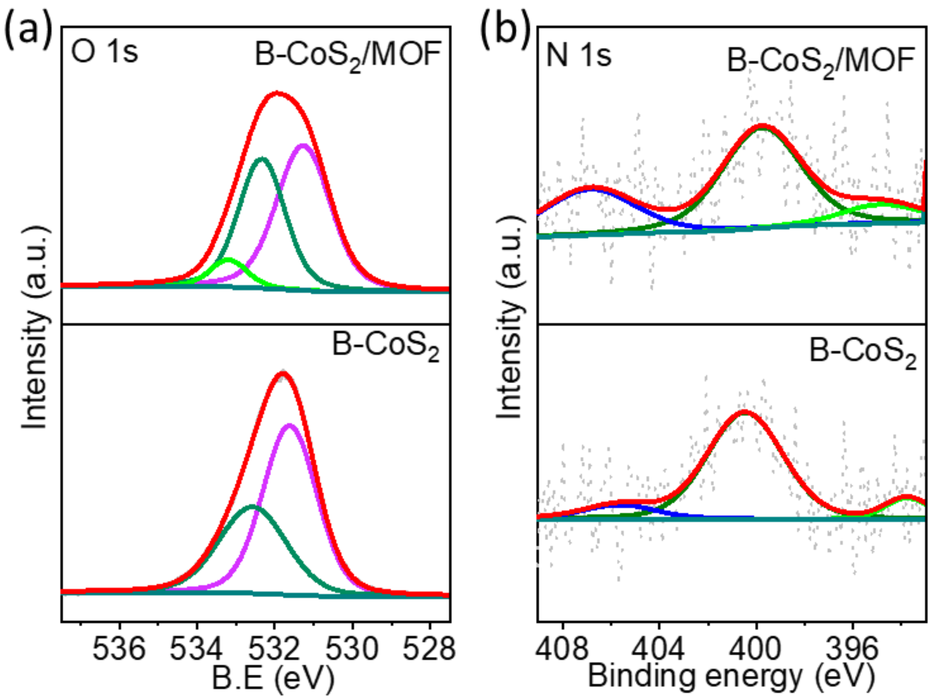

Figure S4. XPS spectra of O 1s, and N 1s of B-CoS_2, B-CoS_2/MOF heterostructure.

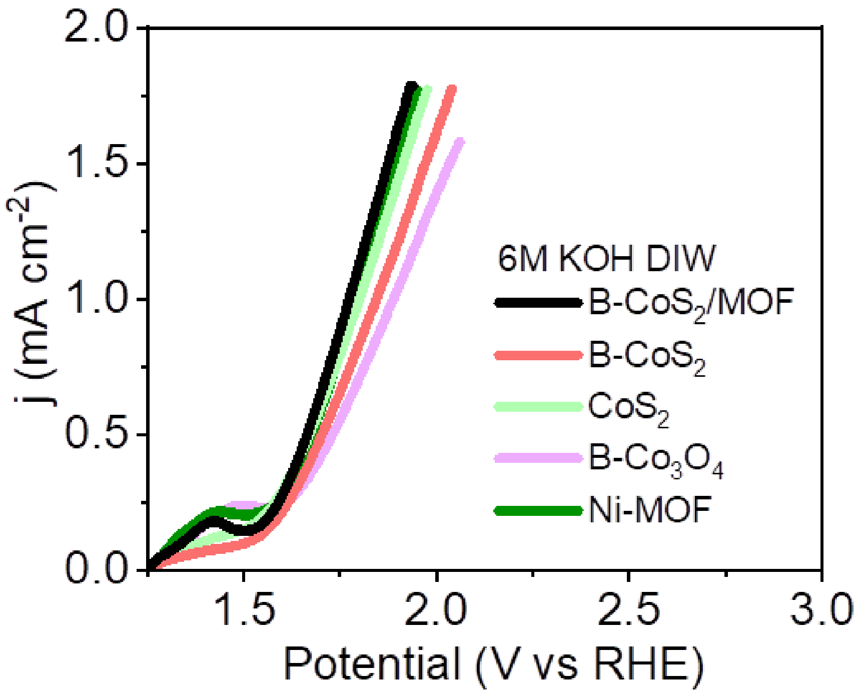

Figure S5. LSV curves of various catalysts in 6 M KOH DI water.

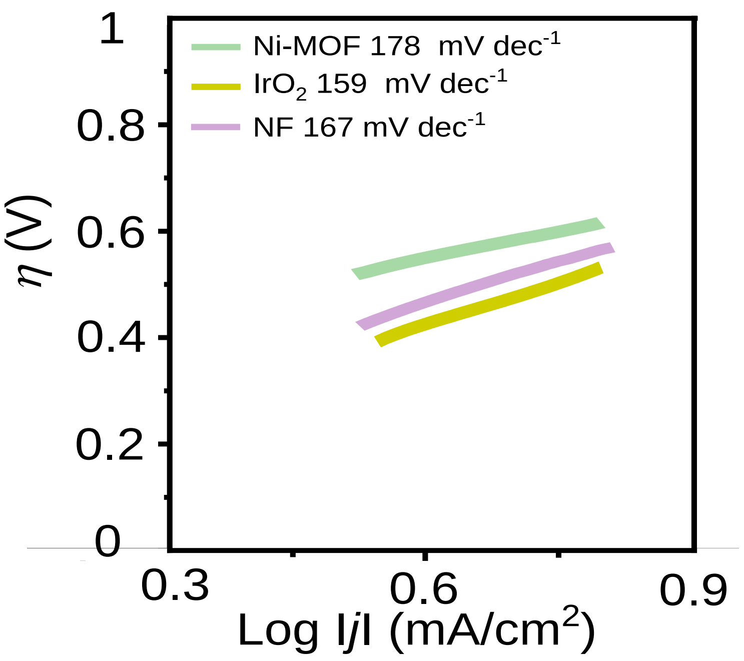

Figure S6. Tafel plots obtained from the corresponding polarization curves.

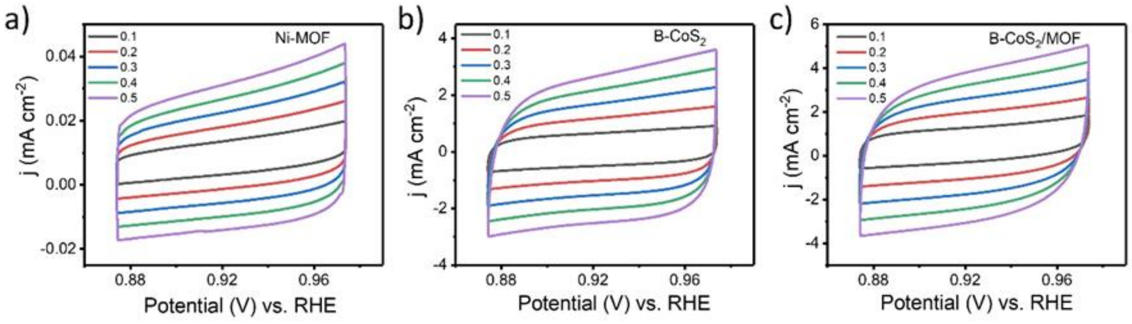

Figure S7. CV curves of Ni-MOF, B-CoS_2, B-CoS_2/MOF heterostructure.

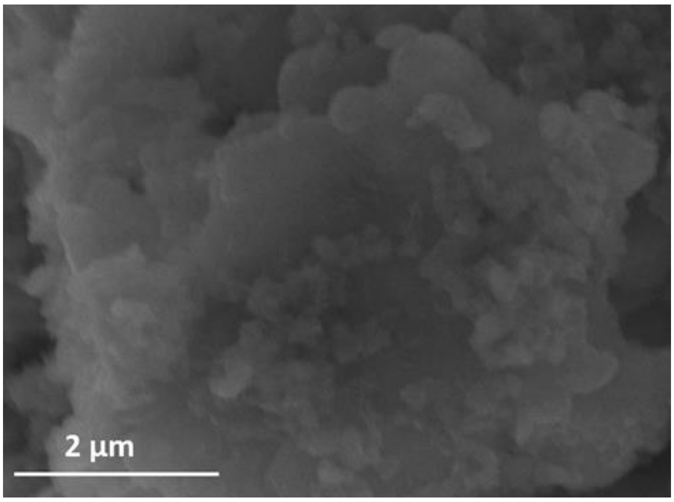

Figure S8. SEM image of the B-CoS_2/MOF heterostructure after high-current-density electrochemical testing.

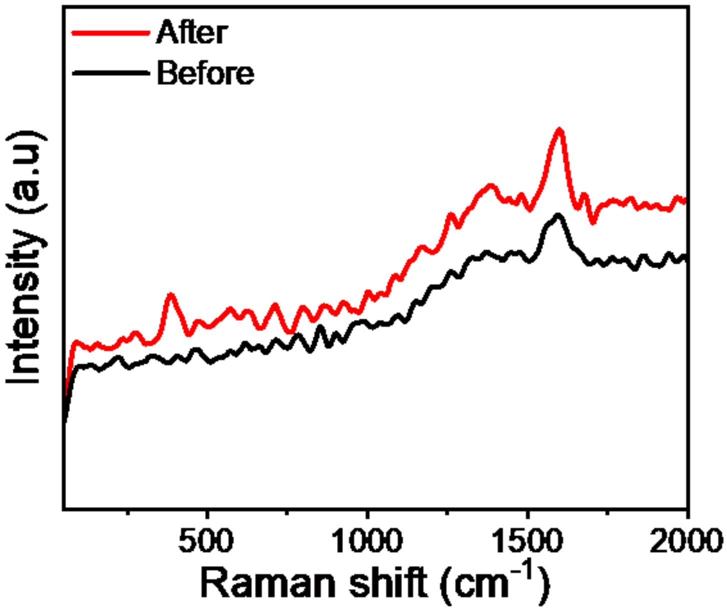

Figure S9. Raman Spectra before and after electrochemical testing

Supplementary Table

Table S1. Comparison of the OER performance of B-CoS_2/MOF with other reported catalysts

Catalysts	Metals	Electrolyte	Overpotential (η)	Tafel Slope	Durability	Ref
NiFe LDH/MOF	Ni, Fe	1.0 M KOH	196 mV@ 10 mA cm^{-2}	32.5 mV dec^{-1}	100 h	[8]
NiFeZr LDH	Ni, Fe, Zr	1.0 M KOH	182 mV@ 10 mA cm^{-2}	38.6 mV dec^{-1}	750 h	[9]
RuO_2/MoO_3	Ru, Mo	0.5 M H_2SO_4 (acid)	167 mV@ 10 mA cm^{-2}	65 mV dec^{-1}	300 h	[10]
CoS_2/MoS_2	Co, Mo	1.0 M KOH	283 mV @ 10 mA cm^{-2}	105.3 mV dec^{-1}	24 h	[11]
FeMn-MOF	Fe, Mn	1.0 M KOH (seawater)	255 mV @ 100 mA cm^{-2}	49.6 mV dec^{-1}	500 h	[12]
Mo-NiFeOOH	Mo, Ni, Fe	6 M KOH (seawater)	316 mV@2000 mA cm^{-2}	16.6 mV dec^{-1}	200 h	[13]
$Ru_{0.1}Mn_{0.9}O_x$	Ru, Mn	0.01 M $HClO_4$ (seawater)	211 mV@10 mA cm^{-2}	62.8 mV dec^{-1}	1200 h	[14]
Ru0.1-NiFeOOH/PO_4^{3-}	Ru, Ni, Fe	6 M KOH (seawater)	230 mV@10 mA cm^{-2}	62.8 mV dec^{-1}	1000 h	[15]
**B-CoS_2/MOF**	**Co, Ni**	**6 M KOH (seawater)**	**542 mV@ 1000 mA cm^{-2}**	**69 mV dec^{-1}**	**600 h**	**This work**

Table S2. Corrosion testing of different electrodes in 5% NaCl DI water electrolyte.

Catalysts	E_{corr} (V)	I_{corr} (mA cm^{-2})	Corrosion rate (cm $year^{-}$)

B-CoS_2/MOF	-0.41	0.212	0.848
B-CoS_2	-0.48	0.219	0.876
CoS_2	-0.67	0.224	0.896
Ni-MOF	-0.58	0.216	0.864

Supplementary Note 1.

Faradaic Efficiency calculations

Faradaic efficiency: (experimental moles of O_2 gas/theoretical moles of O_2 gas) * 100. Where the theoretical amount of O_2 gas was calculated using the equation:

$$n = \frac{I \times t}{z \times F}$$

where, n= no. of moles of O_2, I= current (A), t= time (s), z= transfer of electrons (z= 4 for O_2), F= Faraday constant (96500 C/mol).

At the lab temperature of 17 °C,

P_{water} = 14.53 mm Hg (chosen from the vapour pressure table).

P_{oxygen} = 762 – 14.53 = 747.47 mm Hg or 0.98 atm. The no. of oxygen gas moles produced in displacing water can be calculated by the equation:

$$N = \frac{PV}{RT}$$

Where V= 32.4 ml, volume of gas produced,

T= 290 K, R= ideal gas constant (0.0821 L atm/mol K),

N= 1.44 mmoles

n=1.4 mmoles

t=300 s, volume 20mmhg

Faradaic efficiency = (N/n) *100 = 91 %

References

[1] P.E. Blöchl, Physical Review B, 50 (1994) 17953.

[2] G. Kresse, J. Furthmüller, Physical Review B, 54 (1996) 11169.

[3] G. Kresse, D. Joubert, Physical Review B, 59 (1999) 1758.

[4] J.P. Perdew, K. Burke, M. Ernzerhof, Physical Review Letters, 77 (1996) 3865.

[5] O.Y. Long, G. Sai Gautam, E.A. Carter, Physical Review Materials, 4 (2020) 045401.

[6] S. Grimme, J. Antony, S. Ehrlich, H. Krieg, The Journal of Chemical Physics, 132 (2010) 154104.

[7] V. Wang, N. Xu, J.-C. Liu, G. Tang, W.-T. Geng, Computer Physics Communications, 267 (2021) 108033.

[8] H. Yin, S. Su, D. Yao, L. Wang, X. Liu, T. T. Isimjan, X. Yang, and D. Cai, *Inorganic Chemistry Frontiers*, 11 (2024) 2489.

[9] R. Zhao, S. Xu, D. Liu, L. Wei, S. Yang, X. Yan, Y. Chen, Z. Zhou, J. Su, and L. Guo, *Applied Catalysis B: Environmental*, 338 (2023) 123027.

[10] W. Gou, S. Zhang, Y. Wang, X. Tan, L. Liao, Z. Qi, M. Xie, Y. Ma, Y. Su, and Y. Qu, *Energy & Environmental Science*, 17 (2024) 6755.

[11] Z. Li, Q. Ma, S. Zhang, D. Zhang, H. Wang, Q. Wang, H. Sun, and B. Wang, *New Journal of Chemistry*, 48 (2024) 6688.

[12] R. Yuan, C. Liao, L. Cao, D. Li, S. Sun, G. Wang, G. Li, J. Xie, and Z. Shao, *Advanced Functional Materials*, (2025) e08413.

[13] L. Yu, L. Chi, Y. Wang, M. Ning, E. Nasr, S. Song, J. Luan, S. Chen, and Z. Ren, *ACS Energy Letters*, 10 (2025) 5664.

[14] J. Xu, C. C. Kao, H. Shen, H. Liu, Y. Zheng, and S. Z. Qiao, *Angewandte Chemie*, 137 (2025) e202420615.

[15] H. Wang, N. Jiang, B. Huang, Q. Yu, and L. Guan, *EES Catalysis*, 2 (2024)1092.
